# Spiritual Well-Being and Associated Factors in End-Stage Renal Disease

**DOI:** 10.1155/2021/6636854

**Published:** 2021-04-30

**Authors:** Evangelos C. Fradelos

**Affiliations:** Nursing Department, University of Thessaly, Larisa, Greece

## Abstract

People with CKD depend on religion and spirituality to deal with their chronic illness, and those are essential means of coping for those living with chronic diseases. The present study aims to evaluate ESRD patients' spiritual wellbeing undergoing hemodialysis treatment and to identify critical variables associated with the spiritual wellbeing of those patients. *Methods*. A cross-sectional study was conducted, in which 367 patients undergoing HD participated. Patients were randomly selected from six HD units in various geographical areas of Greece. Data were collected through an anonymous self-completed questionnaire consisting of two parts. The first part contained questions regarding demographic, social, and clinical information such as age, gender, marital status, and duration of dialysis comorbidities. The second part assessed the patients' spiritual wellbeing with the use of the Functional Assessment of Chronic Illness Therapy-Spiritual Well-Being Scale-12. Multivariate analysis was performed to extract predictors or determinants of spiritual wellbeing of hemodialysis patients. *Results*. From the total of the 367 participants, 228 (62.1%) were males and 139 (37.9%) were females, and the mean age was 61.80 ± 15.11. Spiritual wellbeing had a mean value of 30.55 (SD = 8.22), which means that patients had a satisfactory spiritual wellbeing level. Multivariate analysis revealed that place of residence, marital status, educational level, and comorbidities could predict spiritual wellbeing in ESRD patients. *Conclusions*. There is much evidence in the literature supporting the positive effect of spirituality, health (physical and mental), and quality of life. Integration of spiritual wellbeing evaluation and spiritual care in everyday practice as a part of clinical care can increase the quality of the provided care and improve health outcome for patients undergoing hemodialysis.

## 1. Introduction

Common concerns for human worldwide include questions about life, death, the meaning of existence, and its role in the world. Those questions are usually addressed by religion and spirituality. The World Health Organization considers religion, spirituality, and personal beliefs as important concepts in the evaluation of a person's quality of life [1].

The concept of spirituality is not easy to be determined. Harrison and Bernard argue that there are as many definitions of spirituality as those who are trying to define it. Also, they argue that existing words and definitions are unable to describe the nature of spirituality [2]. In recent years, there has been growing interest in the scientific community, especially the health scientists, in spirituality and its value in treating various disease states [3–6]. It is an indisputable fact that, for many people, spirituality and religiosity are ways to manage the stress and the various difficulties of everyday life. Furthermore, researchers ascribe to spirituality the ability to help people give meaning to difficulties, illnesses, and even death. It is the last moment when people attempt to get out of the realm of uncertainty and gain a sense of wholeness. It is precisely this capacity of individuals to continuously search for healing and fulfilment, which Puchalski (2004) ascribes to spirituality as a human aspect [4].

## 2. Literature Review

The word spirituality comes from the word “spirit,” which means breath, wind, life, intelligence, soul, meaning, and essence, while the concept of spirituality is defined as “the qualities that make up the spiritual level of the person” [7]. According to the Oxford English Dictionary [8], the English words “spirituality” and “spirit” have their common origin in the Latin word “spiritus,” which means “breath of life” [9]. In both cases, the word spirit refers to an individual's non-physical state and to all person's emotions and characteristics, elements that transcend death and physical decay. In a recent study, a person's spirituality is defined as “a human capacity involving and engaging deep feelings and beliefs, especially of a religious nature rather than the physical parts of life” [8].

As an emerging research issue, a lot of researchers tried to define spirituality resulting in many different definitions varying based on the science represented by the researcher who each time tried to define it [9]. Thus, we can conclude to spirituality's complexity, depth, and fluidity, making it clear that one of its key characteristics is that it is experienced, observed, and described, but it is very difficult for individuals to convey its meaning [10]. Spirituality is defined as the relationship with the spirit, and it is perceived to be something greater than the human nature. Spirituality involves humans' search for meaning and purpose in life, namely, that part of the human soul struggling for metaphysical values, concepts, and experiences. Spirit is that aspect or essence of a person that gives him energy and strength and motivates the pursuit of virtues such as love and care. Spirituality encompasses a type of linkage between human and some higher power, either divine or some other form of inner strength [2].

People with CKD depend on religion and spirituality to deal with their chronic illness, which also affects their quality of life. In the context of chronicity, quality of life is a multidimensional, many-sided, dynamic, and subjective view of various degrees of satisfaction related to health. This health-related satisfaction is linked to spiritual wellbeing. Therefore, spirituality or religiousness is an important means of coping for those living with chronic diseases [11]. Spirituality is what gives an overarching meaning to one's life, illness, and death and exists inside and outside traditional religious systems. In contrast, religion is defined more closely and can be seen as the participation in the institutionally accepted beliefs and activities of a particular religious group. It is the external expression or practice of a particular spiritual understanding or framework for belief systems, values, codes of conduct, and rituals. Nowadays, it is recognized that spirituality is pivotal for patients' lives, especially in the context of weakness, suffering, and death, as it provides an interpretive framework for addressing the challenges of the disease [12].

Studies examining spirituality in patients with CKD have concluded that these patients have a number of mental needs that are related to and affect the psychological adjustment to the disease and that these needs appear to remain the same throughout the disease continuum [13]. According to Eslami et al. (2014), religious beliefs and mental health during the course of the disease are among the most important issues, as in times of crisis, or when other coping mechanisms are not effective enough, people become more religious [14]. In these cases, as a strategy and way of life, religious beliefs have been a good source of support for individuals and have equipped them with a variety of effective coping skills [6]. In particular, increased religiousness is one of those mechanisms that allow the search for meaning in life and reduce despair. A coping strategy, inherently linked to religion, is hope, as it leads the individual to act and move according to the prescribed treatment goals. Lack of hope can leave the person without perspective and the person may passively expect to die. Although hope does not have the power of healing, it does encourage the patient to continue to struggle and seek clinical improvement [15].

In Greece, for many individuals, religion and spirituality are important aspects of their sickness and health. Patients are engaged in various religious practices even during hospitalization. A great percentage attribute health and illness in their relation to God [16–18]. In addition, many religious and spiritual practices are driving lifestyle habits such as fluids and diet (fasts) that are directly linked to hemodialysis outcomes [19]. To the best of our knowledge, there are no studies in Greece which investigate predicting factors of spiritual wellbeing among HD patients. Therefore, the purpose of the present study is to investigate social, demographic, and clinical factors related to the spiritual wellbeing of patients with chronic kidney disease undergoing dialysis.

## 3. Methodology and Research Design

A cross-sectional, correlational study design was used in this study. The study sample consisted of patients with end-stage kidney disease who underwent hemodialysis.

### 3.1. Sample, Setting, and Recruitment

The study was conducted in 2018 in a private HD unit named “Iatriko Therapeutirio Iliou Medifil A.E.” (Athens) and in five HD units of public hospitals: (i) General Hospital of Lamia (Central Greece), (ii) Panarkadiko General Hospital “Evangelistria” (South Greece), (iii) General Hospital of Chios Island “Skylitseio” (East Greece), (iv) General Hospital of Athens “G. Gennimatas” (Athens), and (v) University Hospital of Alexandroupolis (North Greece) were randomly selected from each one. The sample consisted of 367 patients undergoing HD who were randomly selected from six HD units in Greece's various geographical areas. The inclusion criteria were the following: (i) age above 18, (ii) undergoing HD 3 times/week for at least 6 months, (iii) native language-Greek, (iv) ability to read and sign the consent form, (v) time-space oriented, and (vi) not currently undergoing transplant procedures. Patients who have been diagnosed with mental or cognitive disorders (according to medical records) were excluded from the study.

### 3.2. Ethics

To carry out the study, licenses were obtained from the Data Protection Authority (protocol number: ΓΝ/ΕΞ/4670-3/04-08-2016), and Scientific Councils of the six HD units. In all cases, oral and written information was provided to patients about the aims of the work, the confidentiality, and anonymity of the answers and their right to interrupt at any time during the procedure.

### 3.3. Data Collection Method-Measurement Tools

Data were collected through an anonymous self-completed questionnaire consisting of two parts:

- The first part contained questions regarding demographic, social, and clinical information such as age, gender, marital status, and duration of dialysis comorbidities. Moreover, some additional information was gathered, such as self-reported religiosity using a single item religiosity “How religious are you?” with a five-point Likert scale ranging within 0–4, where 0 corresponded to not religious and 4 to highly religious. “How close do you feel to God?” was a single item assessing connection to God in a five-point Likert scale ranging within 0–4, where 0 corresponded to not close at all, while 4 corresponded to a close as I can be. Finally, “current activity level” was assessed in a four-point Likert scale ranging within 0–4, where 0 = normal activity, without symptoms, 1 = some symptoms, but do not require bed rest during waking day, 2 = require bed rest for less than 50% of waking day, 3 = require bed rest for more than 50% of waking day, and 4 = unable to get out of bed.
- Functional Assessment of Chronic Illness Therapy-Spiritual Well-Being Scale (FACIT-Sp-12). This is a scale created by Cella et al. [20] and has been widely used to assess spirituality in chronic patients. It is part of a larger assessment tool that measures important functionality factors in patients with chronic disease. Specifically, it includes three subscales: meaning in life, harmony and peace, and the sense of support and power that comes from faith. Each factor of spirituality includes 4 questions in five-point Likert scale with 0 representing the “not at all” answer and 4 representing the “very much.” The questions refer to the last 7 days. Higher scores represent greater spiritual wellbeing. The sum of all the answers gives information about the general spiritual wellbeing. This is a valid tool with a high reliability index (Cronbach's alpha 0.87). The FACIT-sp-12 has been translated and validated for the Greek population in a previous study by Fradelos et al. [21]. The scales' internal consistency reliability in this study was assessed with the Cronbach's alpha reliability coefficient. Cronbach's alpha coefficient values were *α* = 0.82 for the FACIT-Sp12 Spirituality Scale.

### 3.4. Statistical Analysis

The statistics of the research's empirical data were processed with SPSS v. 22.0 for Windows (SPSS, Inc., Chicago, IL, USA). Descriptive and inferential statistical methods were generated. Continuous variables were presented with mean, standard deviation, and range (min-max), while categorical variables were presented as absolute (*n*) and relative (%) frequencies. Scores on FACIT-Sp12 Scale were used as the outcomes (dependent variables) and patient characteristics as the determinants (independent variables). Initially, we performed bivariate analyses. Student's *t*-test and one-way analysis of variance were used for the association between categorical and continuous variables and Pearson's correlation coefficient for the correlation between continuous variables. Multivariate linear regression analysis (stepwise method) was applied to identify predictors of spiritual wellbeing. Stepwise regression is considered to be the appropriate approach as we want to determine predictors of spiritual wellbeing among sociodemographic and clinical characteristics. Stepwise regression is a step-by-step iterative construction of a regression model. During the model's construction, independent variables to be used in a final model are selected and are added or removed in succession and testing for statistical significance after each iteration. The selection of the variables that will be included in the stepwise procedure is based on the bivariate analyses. Variables that were found to be associated with spiritual wellbeing at a significance level *p* < 0.250 were entered in the procedure [22].

Regression coefficients (*β*) with standard errors and 95% confidence intervals were computed. All reported *p* values were two-tailed, and the statistical significance level was set at 0.05.

## 4. Results

### 4.1. Characteristics of the Sample

Table 1 presents the distribution of the 367 patients undergoing dialysis regarding their sociodemographic and clinical characteristics.

62.1% of the patients in the sample were men, and 37.9% were women. Their age ranged from 18 to 92 years with an average value of 61.80 years (SD = 15.11). The majority (46.9%) were in the age range of 60–79 years. Regarding the place of their permanent residence, 67.3% lived in an urban area, 15.3% in a semi-urban area, and 17.4% in a rural area. Most patients were married (59.1%) and had 1 to 2 children (51.5%). About 3/4 of the patients did not live alone but with another or others (76.6%). In terms of their educational level, the majority were graduates of secondary education (43.6%) while a relatively small percentage of higher education (17.2%). 61.0% of patients were retired, 10.9% self-employed, 8.2% civil or private employees, and 19.9% unemployed or stay-at-home moms. Regarding religion, the vast majority were Orthodox Christians (95.9%). Self-reported religiosity was found to be 2.56 ± 1.08, connection with God score was 2.54 ± 1.11. When asked about how religious they are and how close they feel to God, the majority (i.e., 65.1% and 62.7%, respectively) said “somewhat” or “a lot” on the five-point scale that was used. The years undergoing dialysis ranged from 1 to 26 with an average value of 5.69 years (SD = 5.25), while the majority (62.9%) was in the range of 1–5 years. 52.6% of the patients had another health problem. Regarding the current level of activity and the burden of symptoms in everyday life, 31.3% of patients stated that it was at a good level, 29.7% at a moderate level, 19.9% at a bad or very bad level, and 19.1% at a very good level. Finally, the mean score on current activity level of the patients was 2.46 ± 1.09.

### 4.2. FACIT-Sp12 Scale

Table 2 presents the descriptive statistical measures of FACIT-Sp12 Scale and of its subscales.

The Total FACIT-Sp-12 scores (Total Spirituality) ranged from 3 to 47 with an average value of 30.55 (SD = 8.22) and a mean of 31.00. Both the mean and median values were greater than the value 24 corresponding to the midpoint of the response measurement scale (theoretical index range), indicating that the majority of patients had relatively high total spirituality values.

The mean value of the individual dimensions of the scale was for “meaning” 11.99 (SD = 3.27), “peace” 9.26 (SD = 3.38), and “faith” 9.30 (SD = 3.95). All dimensions of spirituality showed mean value and median above 8, which corresponds to the middle point of the scale of measuring the answers (theoretical range of values). Comparatively, the highest mean value was of the “meaning” and the lowest was “peace” with “faith.”

### 4.3. Predictors of Spirituality (Multivariate Analysis)

A multivariate analysis was performed to identify predictors or determinants of spirituality (FACIT-Sp12 Scale) of patients undergoing dialysis. In the multivariate analysis, the characteristics of the patients related to spirituality were introduced at the significance level of 0.25 from the bivariate analysis.  Table 3 presents the multivariate analysis using the statistical model of multiple linear regression with the method of stepwise integration of variables.

From the statistical multivariate analysis for the extraction of predictors of spirituality (“FACIT-Sp12 Scale”), from the characteristics of the patients, the following were found.

Place of residence, marital status, educational level, how close they feel to God, and current level of activity have emerged as predictors of the “meaning” dimension. The statistical model's reported variables explain about 22% of the variability of “meaning” (corrected *R*^2^ = 22.3%). Particularly, the mean value of “meaning” was 1.399 points lower for those living in non-urban areas than for those living in urban areas (*β* = −1.399 *p* < 0.001). The mean value of “meaning” was 1.049 points lower in the unmarried than in the married (*β* = −1.049 *p* = 0.001). The mean value of “meaning” was by 1.118 points and 1.494 points, respectively, higher in the graduates of secondary and tertiary education in relation to the graduates of primary education (*β* = 1.118 *p* = 0.001 and *β* = 1.494 *p* = 0.001). Moreover, a change in the estimate of how close they feel to God by 1 point causes a similar change in “meaning” by 0.514 points (*β* = 0.514 *p* < 0.001) and change of the current activity by one level causes a similar change of the “meaning” by 0.741 units (*β* = 0.741 *p* < 0.001).

Marital status, educational level, how close they feel to God, the existence of another health problem, and the current level of activity emerged as predictive factors of the “peace” dimension. The statistical model's mentioned variables explain about 23% of the variability of “peace” (corrected *R*^2^ = 22.7%). More specific the mean value of “peace” was 0.919 points lower in the unmarried than in the married (*β* = −0.919 *p* = 0.005). The mean value of “peace” was by 1.703 points and 1.369 points, respectively, correspondingly lower in the graduates of primary and secondary education in relation to the graduates of higher education (*β* = −1.703 *p* < 0.001 and *β* = −1.369 *p* = 0.002). Furthermore, a change in the estimate of how close they feel to God by 1 point causes a similar change in “peace” by 0.778 points (*β* = 0.778 *p* < 0.001). The mean value of “peace” was 0.682 points higher in those who do not have any other health problems than those who have (*β* = 0.682 *p* = 0.041). A change of the current activity by one level causes a similar change of the “peace” by 0.819 points (*β* = 0.819 *p* < 0.001).

The place of residence, how religious they are, how close they feel to God, and the current level of activity have emerged as predictors of the “faith” dimension. The statistical model's mentioned variables explain about 38% of the variability of “faith” (corrected *R*^2^ = 37.7%). Particularly, the average value of “faith” was 0.690 points lower for those living in a non-urban area compared to those living in an urban area (*β* = −0.690 *p* = 0.048). A change in the estimate of how religious they are by 1 unit causes a similar change in “faith” by 0.855 units (*β* = 0.855 *p* = 0.002), and a change in the estimate of how close they feel to God by 1 point causes a similar change in “faith” by 1.420 points (*β* = 1.420 *p* < 0.001). Finally, one level change in current activity causes a similar change in “faith” by 0.343 points (*β* = 0.343 *p* = 0.023).

In addition, the place of residence, the marital status, the educational level, how close they feel to God, and the current level of activity have emerged as predictive factors of “total spirituality.” The statistical model's mentioned variables explain about 36% of the variability of “total spirituality” (Corrected *R*^2^ = 36.1%). Particularly, the mean value of “total spirituality” was 2.372 points lower for those living in a non-urban area than for those living in an urban area (*β* = −2.372 *p* = 0.002). The average value of “total spirituality” was 2.493 points lower in the unmarried than in the married (*β* = −2.493 *p* = 0.001). The mean value of “total spirituality” was by 3.237 points and 2.482 points, respectively, lower in the graduates of primary and secondary education in relation to the graduates of higher education (*β* = −3.237 *p* = 0.002 and *β* = −2.482 *p* = 0.013). In addition, a change in the estimate of how close they feel to God by 1 unit causes a similar change in “total spirituality” by 3.337 units (*β* = 3.337 *p* < 0.001) while a change in current activity by one level causes a similar change in “total spirituality” by 1.995 points (*β* = 1.995 *p* < 0.001).

## 5. Discussion

Exploring and evaluating patients' religious and spiritual beliefs undergoing renal replacement therapy contributes to the design and delivery of personalized care based on patients' preferences. Studies show that the integration of these beliefs and the provision of this type of care is associated with increased patient satisfaction with life and increased levels of social support [23]. According to the results of the present study, Greek patients undergoing dialysis have lower levels of spirituality in comparison to the results of similar studies in foreign patients with CKD [13], but also in comparison to other categories of patients, such as women who have been diagnosed with breast cancer [24, 25]. However, the present study results are similar to those in patients with diabetes mellitus [24].

Regarding the factors related to spirituality, the study results show that important factors are, among others, gender, age, and comorbidity. Nevertheless, in the predictive regression model, one observes that the place of residence, the connection with God, the educational level, and also the burden of functionality due to the symptoms are the most frequently introduced in the regression models.

The educational level was found to be associated with high levels of spirituality, a finding that comes to reinforce the already reported results derived from the international literature. In addition, it is widely accepted that, in times of crisis, such as the diagnosis of chronic, life-threatening, or end-stage disease, people tend to be more spiritual in trying to give meaning to the illness and pain they experience [26, 27]. The present study also showed that most dimensions of spirituality are positively related to functionality (*p* < 0.001). This finding is in complete agreement with the modern literature data, which demonstrates the beneficial effect of spirituality on the general state of health [26].

Another factor that has been found to be a predictor of high levels of spirituality is the connection with God, that is, feeling close to the divine. Spirituality and religiousness can be two different concepts, as already mentioned, yet there is a close connection between them. A person may be spiritual without being religious, but a person who is religious certainly has high levels of spirituality [28]. Belavich and Pargament (2002) [29] studied the relationship between spirituality and the connection with God in individuals who had a familiar person in the operating room. In this study, it was found that there was a strong relationship between these two variables and more specifically with the positive mental treatment of difficulties in particular. These findings confirm that spirituality is a source of strength and courage [30].

Finally, from the clinical factors, only comorbidity was found to be a predictor in the multivariate model and only in the “peace” dimension of spirituality. This finding is in line with studies that want patients with many health problems to turn to religion and spirituality for support. A study in Australia involving men and women aged 55–85 found that spirituality and participation in religious activities play a beneficial role in receiving social support and can help people manage the presence of multiple diseases in their lives better [31].

## 6. Conclusion

For many people, religion and spirituality are central pillars in their lives. These axes should be considered when designing care provision for these patients, as there is a lot of evidence in the literature to support this fact. The values obtained by both the overall spirituality scale and the individual dimensions of meaning, peace, and faith are above average, leading to the conclusion that the patients' spirituality levels in the present study were moderate to high. Taking this into account and relating this finding to Lazarus and Folkman's [32] transactional theory of stress and coping, the finding of strong correlations between the various parameters is partially explained. The place of residence, marital status, multiple health problems, and also the educational level were found to be related to the level of spiritual wellbeing of the patients with CKD who undergo dialysis. These findings could provide guidance for planning, designing, and implementing interventions to improve these patients' spiritual wellbeing since, as highlighted by the literature, the increased levels of spiritual wellbeing can lead to a better quality of life and less mental strain.

## Figures and Tables

**Table 1:** Characteristics of the sample (*n* = 367).

Characteristics	*n*	%
Gender		
Male	228	62.1%
Female	139	37.9%

Age (years)		
≤39	37	10.1%
40–59	113	30.8%
60–79	172	46.9%
≥80	45	12.3%
Mean ± SD	61.80 ± 15.11	
Range	18–92	

Place of residence		
Rural area	64	17.4%
Suburban area	56	15.3%
Urban area	247	67.3%

Marital status		
Unmarried	69	18.8%
Married	217	59.1%
Divorced	31	8.4%
Widowed	50	13.6%

Number of children		
0	108	29.4%
1–2	189	51.5%
≥3	70	19.1%
Mean ± SD	1.57 ± 1.41	
Range	0–10	

Do you live alone?		
Yes	86	23.4%
No	281	76.6%

Educational level		
Some classes of elementary school	28	7.6%
Elementary school graduate	116	31.6%
High school graduate	160	43.6%
University graduate	63	17.2%

Professional status		
Unemployed	37	10.1%
Household	36	9.8%
Self-employed	40	10.9%
Private employee	15	4.1%
Civil servant	15	4.1%
Retired	224	61.0%

Religion		
Christian Orthodox	352	95.9%
Christian Catholic	0	0.0%
Muslim	6	1.6%
Other	9	2.5%

How religious are you?		
0 = not at all	20	5.4%
1 = a little	37	10.1%
2 = somehow	100	27.2%
3 = much	139	37.9%
4 = very much	71	19.3%

How close do you feel to God?		
0 = not at all	21	5.7%
1 = a little	40	10.9%
2 = somehow	102	27.8%
3 = much	128	34.9%
4 = very much	76	20.7%

Duration of dialysis (years)		
1–5	231	62.9%
6–10	89	24.3%
11–15	25	6.8%
≥16	22	6.0%

Experiencing other health problems?		
Yes	193	52.6%
No	174	47.4%

Current level of activity		
0 = very bad (I cannot get out of bed)	14	3.8%
1 = bad (require bed rest for more than 50% of the day)	59	16.1%
2 = moderate (require bed rest for less than 50% of the day)	109	29.7%
3 = good (some symptoms, but I do not require bed rest during the day)	115	31.3%

**Table 2:** Descriptive statistical measures of FACIT-Sp12 Scale (*n* = 367).

FACIT-Sp12 Scale	Mean ± SD	Median	Range
Meaning (theoretical range: 0–16)	11.99 ± 3.27	13.00	1–16
Peace (theoretical range: 0–16)	9.26 ± 3.38	9.00	1–16
Faith (theoretical range: 0–16)	9.30 ± 3.95	10.00	0–16
Total FACIT-Sp-12 scores (theoretical range: 0–48)	30.55 ± 8.22	31.00	3–47

**Table 3:** Multiple linear regression (stepwise method) with FACIT-Sp12 Scale as a dependent value and patients characteristics as independent variables ^*∗*^ (*n* = 367).

FACIT-Sp12 scale	Predictors	*β*	SE	95% CI	*p* value

Meaning	Constant (*α*)	11.450	0.866	9.747 to 13.153	<0.001
	Area of residence				
	Urban (reference category)	0	—	—	—
	Non-urban	−1.399	0.330	−2.047 to −0.750	<0.001
	Marital status				
	Married (reference category)	0	—	—	—
	Unmarried	−1.049	0.315	−1.669 to −0.428	0.001
	Educational level				
	Primary (reference category)	0	—	—	—
	Secondary	1.118	0.344	0.442 to 1.794	0.001
	University	1.494	0.458	0.593 to 2.395	0.001
	How close to God are you?	0.514	0.139	0.240 to 0.788	<0.001
	Current activity level	0.741	0.146	0.454 to 1.027	<0.001
	*R* ^2^ = 22.3%, *F* = 18.525, *p* < 0.001	—	—	—	—

FACIT-Sp12 Scale	Predictors	*β*	SE	95% CI	*p* value

Peace	Constant (*α*)	6.829	0.956	4.950 to 8.708	<0.001
	Marital status				
	Married (reference category)	0	—	—	—
	Unmarried	−0.919	0.325	−1.558 to −0.281	0.005
	Educational level				
	Primary	−1.703	0.462	−2.612 to −0.794	<0.001
	Secondary	−1.369	0.444	−2.242 to −0.497	0.002
	University (reference category)	0	—	—	—
	How close to God are you?	0.778	0.143	0.496 to 1.060	<0.001
	Other health problems				
	Yes (reference category)	0	—	—	—
	No	0.682	0.333	0.027 to 1.337	0.041
	Current activity level	0.819	0.159	0.506 to 1.132	<0.001
	*R* ^2^ = 22.7%, *F* = 18.897, *p* < 0.001	—	—	—	—

FACIT-Sp12 Scale	Predictors	*β*	SE	95% CI	*p* value

Faith	Constant (*α*)	3.585	0.737	2.135 to 5.035	<0.001
	Area of residence				
	Urban (reference category)	0	—	—	—
	Non-urban	−0.690	0.348	−1.375 to −0.005	0.048
	How religious are you?	0.855	0.269	0.325 to 1.385	0.002
	How close to God are you?	1.420	0.263	0.903 to 1.936	<0.001
	Current activity level	0.343	0.151	0.047 to 0.639	0.023
	*R* ^2^ = 37.7%, *F* = 56.328, *p* < 0.001	—	—	—	—

FACIT-Sp12 Scale	Predictors	*β*	SE	95% CI	*p* value

Total FACIT-Sp-12	Constant (*α*)	26.185	2.057	22.140 to 30.230	<0.001
	Area of residence				
	Urban (reference category)	0	—	—	—
	Non-urban	−2.372	0.752	−3.852 to −0.892	0.002
	Marital status				
	Married (reference category)	0	—	—	—
	Unmarried	−2.493	0.719	−3.907 to −1.078	0.001
	Educational level				
	Primary	−3.237	1.045	−5.292 to −1.182	0.002
	Secondary	−2.482	0.992	−4.432 to −0.531	0.013
	University (reference category)	0	—	—	—
	How close to God are you?	3.337	0.318	2.712 to 3.962	<0.001
	Current activity level	1.995	0.333	1.341 to 2.648	<0.001
	*R* ^2^ = 36.1%, *F* = 35.461, *p* < 0.001	—	—	—	—

Notes: *β* = regression coefficient, SE = standard error, CI = confidence interval. ^*∗*^In the multivariate model, the characteristics of the patients were included in the bivariate analysis with a significance level at 0.25.

## Data Availability

The data are available upon request.
